# Anticancer potential of myricetin bulk and nano forms in vitro in lymphocytes from myeloma patients

**DOI:** 10.1007/s00204-020-02938-5

**Published:** 2020-10-30

**Authors:** Shabana Akhtar, Mojgan Najafzadeh, Mohammad Isreb, Lisa Newton, Rajendran C. Gopalan, Diana Anderson

**Affiliations:** 1 School of Chemistry and Biosciences, University of Bradford, Bradford, UK; 2 School of Pharmacy and Medical Sciences, University of Bradford, Bradford, UK; 3 Bradford Royal Infirmary (BRI), Bradford, UK

**Keywords:** Myricetin, Lymphocytes, Myeloma, Intrinsic-apoptotic proteins, Oxidative stress, P53

## Abstract

Evading apoptosis and chemo-resistance are considered as very important factors which help tumour progression and metastasis. Hence, to overcome chemo-resistance, there is an urgent requirement for emergence of more effective treatment options. Myricetin, a naturally occurring flavonoid, is present in various plant-derived foods and shows antitumour potential in different cancers. In the present in vitro study, results from the comet assay demonstrated that myricetin bulk (10 µM) and nano (20 µM) forms exhibited a non-significant level of genotoxicity in lymphocytes from multiple myeloma patients when compared to those from healthy individuals. Western blot results showed a decrease in Bcl-2/Bax ratio and an increase in P53 protein levels in lymphocytes from myeloma patients, but not in lymphocytes from healthy individuals. A significant increase in intracellular reactive oxygen species level was also observed, suggesting that regulation of apoptotic proteins triggered by myricetin exposure in lymphocytes from myeloma patients occurred through P53 and oxidative stress-dependent pathways. The potency of myricetin against lymphocytes from myeloma patients marks it a potential candidate to be considered as an alternative to overcome chemo-resistance in cancer therapies.

## Introduction

Cancer is a serious threat to human health and life at present that is constantly increasing (Yang et al. 2011). Chemotherapy, steroids, biological therapies and possibly stem cell transplant are the treatment methods currently being utilised for the therapy of multiple myeloma (MM) cancer. Initially, chemotherapy combined with other treatment works effectively, but myeloma patients usually always have a relapse and these drugs also cause various side effects including alopecia, nausea, neuropathy, etc.; therefore, more careful drugs and novel therapies are required for human cancers (Huang et al. 2015).

Cell cycle ensures the homeostasis in an organ, while a dysregulation in any of its steps or components could lead to cancer development. The molecular targets involved in the cell cycle regulatory mechanisms are the main focus of investigational anticancer drugs (Diaz-Moralli et al. 2013).

The maintenance of apoptosis balance is highly essential for normal cellular growth, as excessive apoptosis causes atrophy, whereas faulty apoptosis leads to uncontrolled cellular growth which is implicated in various illnesses including cancer. Hence, inducing apoptosis may be a promising strategy to overcome various problems related to cancer therapies (Hall et al. 2008). Various processes are involved in the inhibition of apoptosis in cancer cells such as P53 mutations and the expression of *P*-glycoprotein (Brunelle and Letai 2009). Mitochondria play a key role in apoptosis and other cellular metabolic processes. When apoptosis is induced, a variety of metabolic signals produced by mitochondria, cytosol and the membrane are triggered by stimuli. These signals can disrupt the energy metabolisms and modify the expression of Bcl-2 family proteins (Seo et al. 2003). Reactive oxygen species (ROS), mainly produced by mitochondria intracellularly, are considered to be the second messenger to participate in cellular mechanisms such as apoptosis and proliferation. The anticancer drugs including Taxol induce apoptosis in cancer cell mediated by increasing intracellular ROS levels (Perkins et al. 2000; Varbiro et al. 2001; Kim et al. 2013).

Myricetin belongs to a class of flavonoids called flavonols, which exhibit antioxidant properties (Ong and Khoo 1997; Semwal et al. 2016) and mainly occur in fruits, nuts, berries, vegetables and red wine (Basli et al. 2012; Pérez-Cano and Castell 2016). Many past studies have demonstrated that myricetin induces apoptosis in various cancer cell lines comprising hepatoma, colon carcinoma cells, oesophageal, ovarian and pancreatic cancer (Phillips et al. 2011; Zhang et al. 2013; Zang et al. 2014; Xu et al. 2016). In the literature, there is no evidence of research on the mechanism of action of myricetin bulk and nanoparticles in lymphocytes of multiple myeloma patients. As MM originates from the plasma cells which are a type of B lymphocytes (Ghosh and Matsui 2009), in our current study we used surrogate cells, lymphocytes, as model cells to examine the in vitro effects of different particle sizes of myricetin, i.e. the bulk (MYR B) and nanoparticle form (MYR N) and also investigated the molecular mechanisms involved in their effects. We further investigate the effects of myricetin on intracellular ROS levels in lymphocytes.

## Materials and methods

### Blood sample collection and ethics

The current project involving the use of human peripheral lymphocytes was granted ethical approval by Leeds East Ethics Committee (IRAS Reference No.:12/YH/0464) and the University of Bradford’s Sub-Committee for Ethics in Research involving healthy Human Subjects (Reference No.: 0405/8). All peripheral blood samples (Tables 1 and 2) were collected after informed consent from patients and healthy individuals. The research support and governance office of Bradford Teaching Hospitals NHS Foundation also agreed to the research (REDA number 1202). Normally for this sort of study if a comet assay is involved, 20 individual patients are compared with a healthy individual group of 20. However, for the current study involving the Western blot technique, three individual patients were compared with three healthy individuals. These numbers are considered statistically acceptable, but we used six individual patients which were available at the time of study.

**Table 1:** Brief information of blood samples from healthy individuals (M, male; F, female)

No	Age	Ethnicity	Gender	Smoking history	Family history
1	60	ASIAN	M	YES	NONE
2	59	CAUCASIAN	F	YES	NONE
3	61	CAUCASIAN	F	NO	NONE
4	52	CAUCASAIN	F	NO	NONE
5	60	CAUCASIAN	F	NO	NONE
6	55	CAUCASIAN	M	NO	NONE

**Table 2:** Brief information of blood samples from MM patients

No	Age	Ethnicity	Gender	Smoking history	Family history	Medical condition
1	55	CAUCASIAN	F	NO	NONE	MULTIPLE MYELOMA
2	56	CAUCASIAN	M	NO	PANCREATIC CACNER	MULTIPLE MYELOMA
3	79	CAUCASIAN	M	NO	BREAST CANCER	MULTIPLE MYELOMA
4	77	CAUCASIAN	F	NO	OVARIAN AND BREAST CANCER	MULTIPLE MYELOMA
5	70	CAUCASIAN	M	NO	NONE	MULTIPLE MYELOMA
6	87	CAUCASIAN	M	NO	NONE	MULTIPLE MYELOMA

### Cell culture and reagent

Lymphocytes from all the samples were isolated and maintained in RPMI-1640 medium (Sigma Aldrich, UK), supplemented with 10% foetal bovine serum FBS (Invitrogen, UK) and 1% penicillin streptomycin (Invitrogen, UK) in a humidified incubator at 5% CO_2_ and at 37 °C. Myricetin was purchased from Sigma Aldrich, UK, and was dissolved in excipient mixture to produce its bulk and nano forms. The particle size of myricetin, stability and constitution of excipient mixture are provided in our previous study (Akhtar et al. 2020a, b). The primary antibodies against P53, Bcl-2, Bax and GAPDH were purchased from Abcam, Cambridge, UK.

### Cell viability

Cytotoxicity was determined by measuring 3-(4,5-dimethylthiazol-2-yl)-2,5-diphenyltetrazoliumbromide (MTT) dye (Sigma Aldrich, UK) absorbance. Lymphocytes (1 × 10^4^) were seeded in 96-well plates and incubated overnight to get attached to the bottom of plates at 37 °C in the presence of CO_2_ 5%. Then cells were treated with different treatment groups for 1, 24 and 48 h. After that media were removed, 10 µl of MTT dye (5 mg/ml) was added to the wells and incubated for 4 h under the same conditions in the dark. Then, formazan crystals were dissolved in 200 µl of DMSO and absorbance was read at 590 nm.

### Determination of ROS production

Cellular ROS was monitored by cellular ROS detection kit using 2′,7′-dichlorofluorescin diacetate (DCFDA) dye (Abcam, UK). Briefly, isolated lymphocytes were seeded overnight in 96-well plates and then treated with chemicals for 1 h. Cells were then washed followed by addition of DCFDA dye into each well and incubated for another 45 min at 37 °C in the presence of 5% CO_2._ Dye was washed off and fluorescence was measured at 485/535 nm.

### DNA damage assessment using the Comet assay

Lymphocytes were treated with MYR B (10 µM) and MYR N (20 µM) for 1 h and the cell suspension was centrifuged at 3000 rpm (1000*g*). The supernatant was removed and the pelleted cells were subjected for the Comet assay as previously defined with minor changes (Singh et al. 1988; Tice et al. 2000; OECD 2016; Anderson et al. 2014; Azqueta and Dusinska 2015).

### Western blot analysis

Lymphocytes were seeded in six-well plates at a concentration of 10^6^cells/well, incubated overnight and treated with chemicals for 24 h. Then cells were lysed and total protein levels were determined using the Bio-Rad Bradford assay kit (Bio-Rad, UK). The cell lysates were separated using protein electrophoresis and blotted on nitrocellulose membrane (Abcam, UK). The membranes were blocked overnight in 5% bovine serum albumin (BSA) diluted in Tris-buffered saline supplemented with 0.1% Tween 20 at 4 °C. The membranes were then incubated with primary and secondary antibody dilutions, overnight at cold and for 1 h at room temperature, respectively.

### Statistical analysis

Results were expressed as mean ± standard error of mean (SEM). GraphPad prism was used to perform statistical calculation. The results were analysed using *t *tests and one-way analysis of variance (ANOVA) to test differences between each treatment and control. A *P* value of < 0.05 was considered statistically significant.

## Results and discussion

### MTT assay

**Fig. 1:**
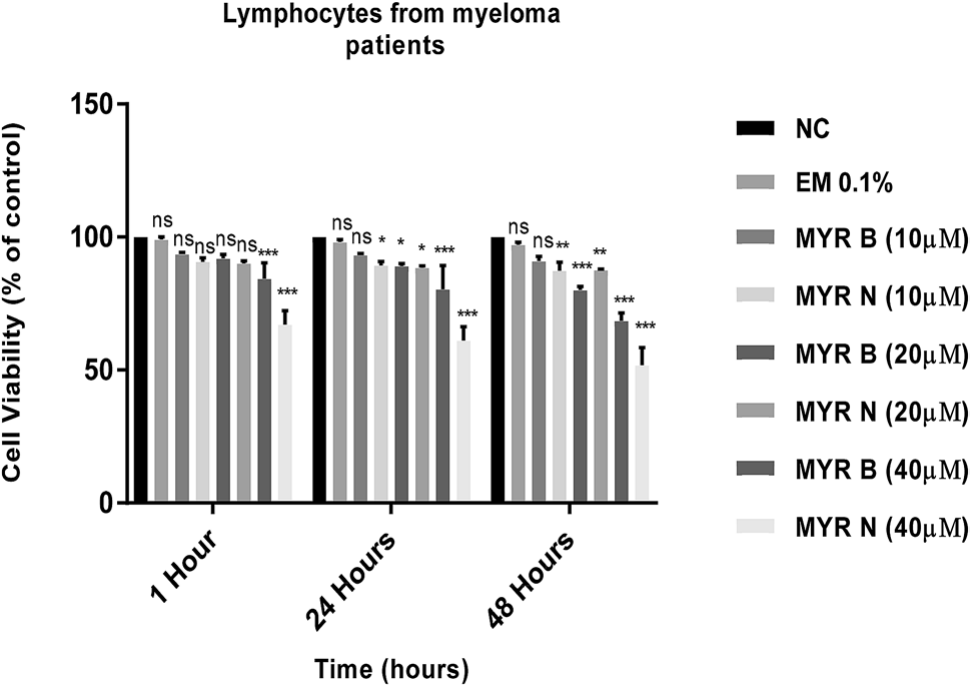
Determination of cell cytotoxicity by MTT-based assay Lymphocytes from MM patients showing cell viability after treatment with different concentrations of MYR B and MYR N. Cell cytotoxicity was expressed as % of the control for 1, 24 and 48 h. The treatment groups included untreated (NC), MYR B (10 µM, 20 µM and 40 µM), MYR N (10 µM, 20 µM and 40 µM) and EM (excipient mixture 0.1%). The best concentrations for MYR B and MYR N were determined by dose response curves. Values are the means of three independent experiments and the error bars represent SDs. (ns, not significant; *P < 0.01; **P < 0.003; ***P < 0.0001)

The effects of MYR B and MYR N on the viability of healthy lymphocytes were compared to those of the untreated group. Neither of the concentrations of MYR B and MYR N reduced viability less than 80% in the healthy group (Akhtar et al. 2020a, b). However, MYR B and MYR N at 40 µM reduced the viability to 68% and 51%, respectively, after 48 h of treatment in lymphocytes from multiple myeloma patients (shown in Fig. 1). Results indicated that cancer cells are more sensitive to myricetin bulk and nanoparticles than the healthy ones, possibly due to the compromised defence and repair mechanisms owing to the disease state. Lower concentrations (10 and 20 µM) of both forms of myricetin did not reduce the viability less than 80% in lymphocytes from the patient group. The effects of the excipient mixture (the vehicle mixture for chemical preparation) were also considered to exclude any errors. Hence, the current study was conducted using the non-toxic concentrations of MYR B and MYR N so that any of the results obtained are not due to the artefact of toxicity. The concentrations of MYR B (10 µM) and MYR N (20 µM) and hydrogen peroxide (H_2_O_2_) (50 µM) used throughout the current in vitro study were determined by dose response studies using the comet assay in our previous study (Akhtar et al. 2020a, b).

### The comet assay

In our previous study (Akhtar et al. 2020a, b), we found no relationship among the confounding factors (age, ethnicity, smoking) regardless of the treatment groups and these factors also do not seem to be contributing in this study towards DNA damage.

**Fig. 2:**
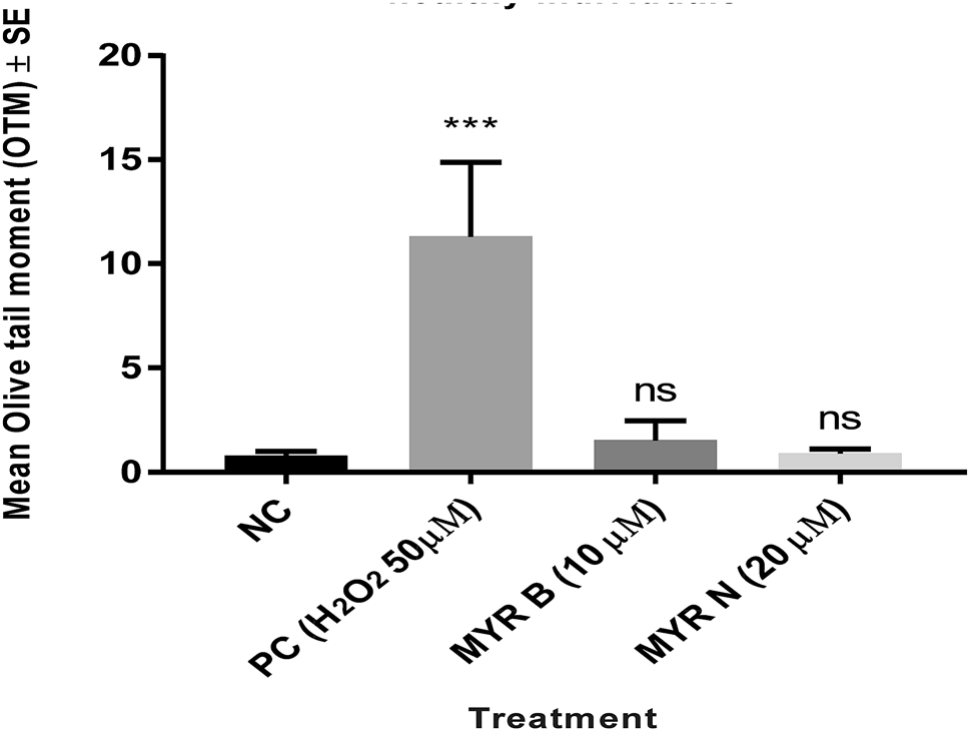
OTM data showing the effect of bulk and nano forms of myricetin on lymphocytes DNA from healthy individuals. The figure shows the mean of experiments in six individuals, counting 100 cells each for four different groups of treatments; an untreated lymphocyte group (NC), positive control (PC) 50 µM H_2_O_2_, myricetin bulk (MYR B 10 µM) and myricetin nano (MYR N 20 µM). All treatment groups were compared to the NC group. (***P < 0.001; ns means not significant analysed by one-way ANOVA. The mean control value was 0.7 and the PC had the maximum mean value of 11.0

**Fig. 3:**
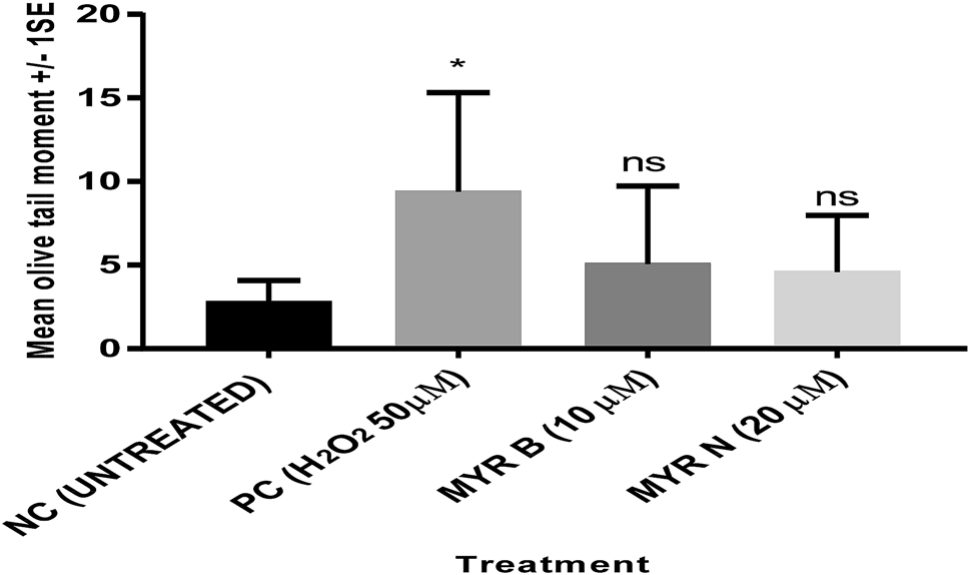
OTM data showing the effect of bulk and nano forms of myricetin on lymphocytes DNA from myeloma patients. The figure shows the mean of experiments in six individuals, counting 100 cells each for four different groups of treatments; an untreated lymphocyte group (NC), positive control (PC) 50 µM H_2_O_2_, myricetin bulk (MYR B 10 µM) and myricetin nano (MYR N 20 µM). All treatment groups were compared to the NC group. The mean control value was 2.7 and the PC had the maximum mean value of 9.3 (*P < 0.001; ns means not significant) analysed by one-way ANOVA

DNA damage and strand break formation caused by MYR B and MYR N in lymphocytes from healthy individuals and myeloma cancer patients were assessed using the comet assay and data were analysed presenting two parameters: % tail DNA and Olive tail moment (OTM). However, due to similar results, only OTM data arew shown. Figure 2 shows no significant effect on DNA damage induced by MYR B and MYR N in healthy lymphocytes. Results from the patient group (Fig. 3) showed a high level of basal DNA damage due to the disease condition and a non-significant genotoxicity induction by MYR B and MYR N treatment.

### Analysis of apoptosis-related proteins using Western blot technique

An investigation was carried out to find whether the mitochondrial dependent intrinsic pathway was involved in apoptosis induction potential of myricetin. The protein expression of major pro-apoptotic and anti-apoptotic protein levels, Bax and Bcl-2, respectively, were analysed using Western blotting.

**Fig. 4:**
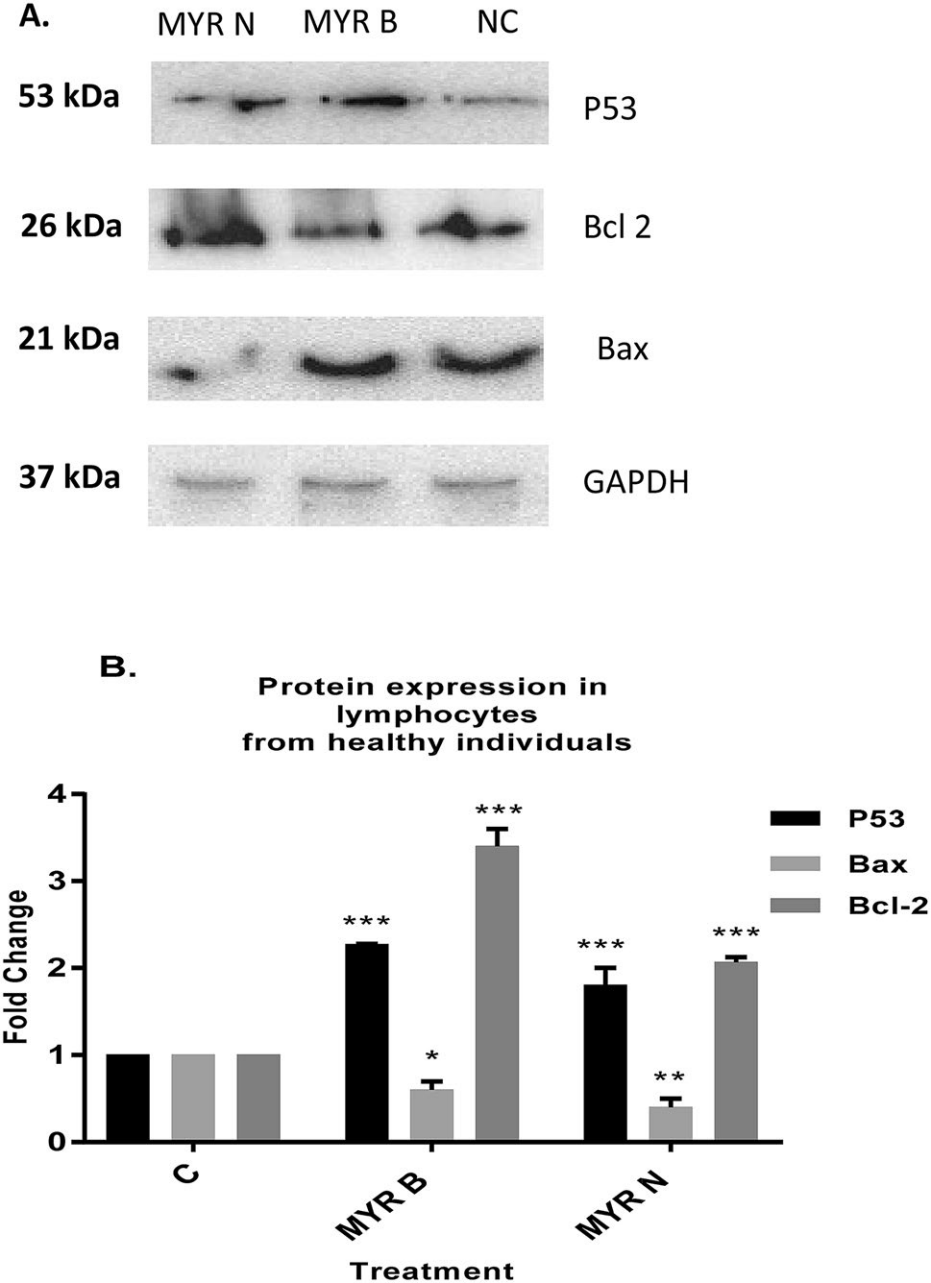
The effect of myricetin bulk and nanoparticles on apoptosis-related proteins in lymphocytes from healthy individuals. **a** Immunoblot analysis of the P53, Bax and Bcl-2 proteins in lymphocyte from healthy individuals treated with MYR B and MYR N. P53 and Bcl-2 expression was increased, while Bax expression decreased. GAPDH was used as an internal control protein to normalise the data. **b** Bar graphs exhibiting fold changes in protein expression levels. Data are represented as the mean ± SE of three experiments. (*P < 0.01; **P < 0.002;, ***P < 0.0001)

**Fig. 5:**
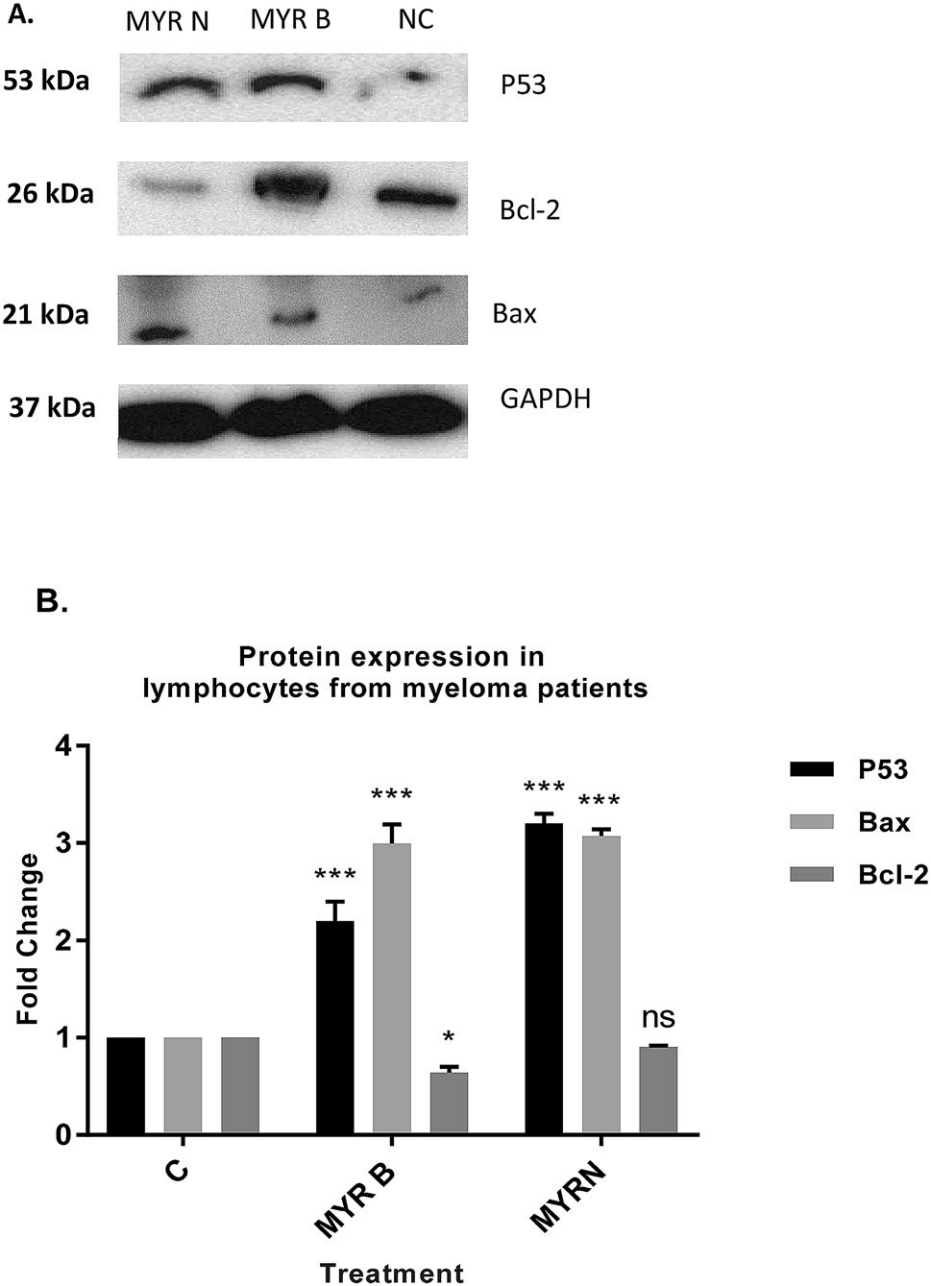
The effect of myricetin bulk and nanoparticles on apoptosis-related proteins in lymphocytes from myeloma patients. **a** Immunoblot analysis of the P53, Bax and Bcl-2 proteins in lymphocyte from MM cancer patients treated with MYR B and MYR N. P53 and Bax expression was increased, whereas Bcl-2 expression was decreased. GAPDH was used as an internal control protein to normalise the data. **b** Bar graphs exhibiting fold changes in protein expression levels. Data are represented as the mean ± SE of three experiments. (*P < 0.01; ***P < 0.0001; ns, not significant)

Results showed (Fig. 4a, b) that Bcl-2 levels were increased by 3.4-fold with MYR B and 2.1-fold with MYR N in lymphocytes from healthy individuals. However, Bax seemed to be down-regulated by 0.6-fold after exposing to MYR B and 0.4-fold with MYR N treatment. In patient lymphocytes, Bax levels were significantly increased by threefold with MYR B and 3.1-fold with MYR N. However, Bcl-2 seemed to be down-regulated, by 0.6-fold after exposing to MYR B and 0.9-fold with MYR N treatment (Fig. 5a, b). These results from Western blot analysis indicated that MYR B and MYR N can potentially induce apoptosis in lymphocytes from myeloma patients by altering the Bcl-2 family proteins expression, but this effect was not evident in lymphocytes from healthy individuals. This might be due to the differential effects of myricetin on healthy and patient lymphocytes. Our results are consistent with a previous study where myricetin induced apoptosis in colon cancer cells by increasing the ratio between Bax and Bcl-2 protein levels (Kim et al. 2014).

P53 is a tumour-suppressor protein which plays a vital role in several cellular processes including apoptosis and angiogenesis (Darcy et al. 2008). In the presence of DNA damage, P53 inhibits the proliferation of damaged cells by different regulatory processes (Haupt et al. 2003; Haupt and haupt 2017). In a number of human cancers, this house-keeper gene is inactivated by mutation leading to unmonitored cell growth. The alterations in the P53 gene are connected with failure in chemotherapy and radiotherapy in various human cancers (Kong et al. 2012). Reintroduction of wild-type P53 into cancer cells, which possess its mutant form, leads to either apoptosis or cell cycle arrest (Martinez et al. 1991; Kim et al. 2013). Increasing the amount of P53 may be a new strategy for treating cancer, as it regulates the intrinsic apoptosis pathway such as Bax (Kuo et al. 2006).

Therefore, we examined P53 protein to verify if myricetin bulk and nanoparticles mediated effects in lymphocytes of myeloma patients through this protein. We found (Fig. 4a, b) that the P53 protein was up-regulated in the lymphocytes of both investigative groups. However its induction was higher in lymphocytes from myeloma patients to 2.2-fold and 3.2-fold after treatment with MYR B and MYR N, respectively. Increased in vitro expression of P53 in lymphocytes, when exposed to myricetin, indicates that myricetin exhibits apoptotic potential.

### Myricetin and ROS production

**Fig. 6:**
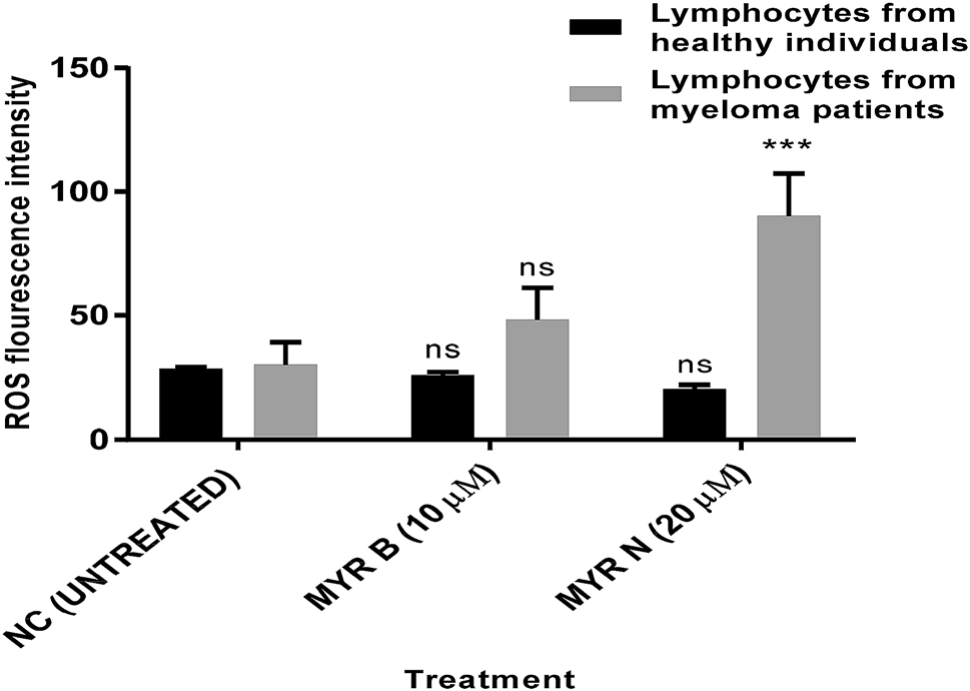
Myricetin-mediated apoptosis is dependent on the production of Reactive oxygen species (ROS) in lymphocytes from MM patients. Isolated lymphocytes from healthy individuals and MM cancer patients were treated with MYR B (10 µM) and MYR N (20 µM) while compared against the respective untreated group (0 µM myricetin). The intracellular ROS levels were assessed using DCFDA assay. Values are the means of three independent experiments and the error bars represent SE, analysed by the two-way ANOVA. (ns, not significant; ***P < 0.001) *P* < 0.05 values were considered significant

A variety of stimuli trigger cytochrome C release and apoptosis through ROS production. However, ROS also play a mitogenic role by inducing proliferation and protecting cells against oxidative stress-induced apoptosis (Kim et al. 2001; Kops et al. 2002; Kim 2017). Hence, data suggest a dual function of ROS. In our study, the mitochondrial-dependent regulation of apoptotic proteins in lymphocytes from myeloma patients was also dependent on ROS production, as ROS levels increased with myricetin bulk and nano-treatment to a significant level when compared to the untreated control and healthy control group (Fig. 6).

## Conclusion

In conclusion, myricetin bulk and nano forms have exhibited anticancer potential and pro-oxidant activities in vitro in lymphocytes from MM cancer patients. It up-regulated the expression of Bax protein while down-regulating Bcl-2 expression. These effects were mediated by P53 and somehow dependent on ROS production.

This is indicative of the potential of myricetin as an anticancer drug for MM. Moreover, MYR N has collectively shown more effective responses than its larger particles (i.e. MYR B) in lymphocytes from both investigated groups.
